# A Novel Test of the Duchenne Marker: Smiles After Botulinum Toxin Treatment for Crow’s Feet Wrinkles

**DOI:** 10.3389/fpsyg.2020.612654

**Published:** 2021-01-12

**Authors:** Nancy Etcoff, Shannon Stock, Eva G. Krumhuber, Lawrence Ian Reed

**Affiliations:** ^1^Department of Psychiatry, Massachusetts General Hospital, Harvard Medical School, Boston, MA, United States; ^2^Department of Mathematics and Computer Science, College of the Holy Cross, Worcester, MA, United States; ^3^Department of Experimental Psychology, University College London, London, United Kingdom; ^4^Department of Psychology, New York University, New York, NY, United States

**Keywords:** facial expression, Duchenne smile, botulinum toxin, emotion, attractiveness

## Abstract

Smiles that vary in muscular configuration also vary in how they are perceived. Previous research suggests that “Duchenne smiles,” indicated by the combined actions of the orbicularis oculi (cheek raiser) and the zygomaticus major muscles (lip corner puller), signal enjoyment. This research has compared perceptions of Duchenne smiles with non-Duchenne smiles among individuals voluntarily innervating or inhibiting the orbicularis oculi muscle. Here we used a novel set of highly controlled stimuli: photographs of patients taken before and after receiving botulinum toxin treatment for crow’s feet lines that selectively paralyzed the lateral orbicularis oculi muscle and removed visible lateral eye wrinkles, to test perception of smiles. Smiles in which the orbicularis muscle was active (prior to treatment) were rated as more felt, spontaneous, intense, and happier. Post treatment patients looked younger, although not more attractive. We discuss the potential implications of these findings within the context of emotion science and clinical research on botulinum toxin.

## Introduction

Among all communicative signals, smiles are one of the most easily recognizable facial expression, with a visual pattern that is detectable over long viewing distances (Smith and Schyns, 2009; Krumhuber et al., 2019). In morphological terms, they can be defined by the activation of the zygomaticus major muscle—a facial muscle which pulls the lip corners upwards and away from the mouth (Ekman, 1989). Despite their simplicity in appearance, smiles can occur in a range of situations and for a variety of reasons. Some smiles express positive emotions such as happiness or enjoyment; hence, they are considered spontaneous readouts of positive internal states (Ekman and Friesen, 1982). Other smiles are deliberately displayed in the absence of an underlying positive affect, for example, to signal politeness, affiliation, or feigned cooperation (Ekman, 1989; Rychlowska et al., 2017). Due to their voluntary nature, those latter expressions are typically described as posed, social, or polite smiles. What is the distinction between spontaneous and posed signals reflecting such disparate functions?

According to some researchers (Ekman, 1992; Frank and Ekman, 1993), the smile of enjoyment is reliably indicated by the contraction of the zygomaticus major muscle with the concurrent contraction of the orbicularis oculi pars lateralis, which is a circumferential muscle surrounding the eye. The latter draws skin toward the eye from the temple and the cheeks, thereby causing narrowing of the eye opening and wrinkles around the eye socket, colloquially called crow’s feet. Activation of both muscles constitutes the so-called “Duchenne smile,” named in homage to the neuroanatomist Duchenne de Boulogne who isolated the orbicularis oculi action and first posited its coherence with enjoyment (Boulogne, 1862/1990; Ekman et al., 1990). In the Facial Action Coding System (Ekman et al., 2002), a system for scoring visible facial movements, the appearance of the Duchenne smile is defined as Action Unit (AU) 6 (orbicularis oculi pars lateralis) coupled with AU12 (zygomaticus major).

The presence of supposedly involuntary eye constriction (AU6) has been proposed to signal happiness/enjoyment in smiles, whereas its absence termed as false, social, non-felt, or non-Duchenne smiles (Frank et al., 1993). While recent studies have revealed that Duchenne smiles occur not only as a spontaneous sign of positive affect and can be deliberately displayed (Schmidt et al., 2006; Krumhuber and Manstead, 2009; Gunnery et al., 2013), there is consistent evidence that Duchenne smiles contribute to perceptions of greater spontaneity and authenticity (Gunnery and Ruben, 2016). They are perceptually salient and perceived as more affectively intense (Malek et al., 2019; Miller et al., 2020), making the smiling person look happier, more amused, and in better humor (Scherer and Ceschi, 2000; Gosselin et al., 2002; Ambadar et al., 2009). Duchenne smiles also lead to favorable interpersonal perceptions (Harker and Keltner, 2001; Messinger et al., 2008), eliciting more positive and affiliative responses in other people (Keltner and Bonanno, 1997) and relieving the concerns of potentially cooperative partners (Reed et al., 2012). Finally, people expressing Duchenne smiles are rated as more likeable, attractive, and intelligent than those showing non-Duchenne smiles (Frank et al., 1993; Quadflieg and Rossion, 2013).

Previous research comparing perceptions of Duchenne and non-Duchenne smiles has been with subjects who had full control of the movements of their facial musculature. Some studies also employed image manipulation techniques by editing the eyes to add/remove visible signs of the Duchenne marker (Calvo et al., 2019; Namba et al., 2020). Here, we compare perceptions of Duchenne and non-Duchenne smiles of subjects before and after botulinum toxin treatment to the orbicularis oculi. Subjects were instructed to display a maximum smile at two time points. In the first, subjects had full control over their facial musculature. In the second, subjects had received botulinum toxin treatment to the orbicularis oculi in order to reduce or eliminate the appearance of crow’s feet lines.

Botulinum toxin is a product of the Clostridium botulinum bacterium that disrupts vesicular exocytosis at neuromuscular junctions producing flaccid paralysis. Very small quantities of formulated pharmaceutical forms of botulinum toxin are injected directly into specific muscles to selectively inhibit activation of the injected muscle. This process is referred to as chemodenervation. In 2016, ASPS (the American Society for Plastic Surgery) reported that there were more than 7 million botulinum toxin procedures performed in the US (American Society of Plastic Surgeons, 2017). One common motivation for seeking botulinum toxin treatment is an individual’s desire to look younger and more attractive. Crow’s feet radiating from the lateral canthus (the outer corner of the eye where upper and lower eyelids meet) is caused by the contraction of fibers of the orbicularis oculi muscle. These wrinkles can appear during expression (dynamic lines) and can become permanent and static with age.

Studies examining the effects of panfacial aesthetic treatment (including botulinum toxin) on observers’ perceptions of patients’ facial characteristics have shown enhancements in physical appearance (Przylipiak et al., 2018). However, to our knowledge, there are no studies investigating these effects following the selective chemodenervation of the orbicularis oculi muscle. The aim of the current study is to examine perceptions of deliberately posed smiles displayed by patients before and after receiving botulinum toxin treatment for crow’s feet lines. It was hypothesized that pre-treatment photographs with no inhibition of the Duchenne marker would be rated as more spontaneous, more intense, and happier than post-treatment photographs in which the lateral orbicularis oculi muscle has been selectively chemodenervated.

## Materials and Methods

### Participants

Three hundred and ninety-three (185 female) participants from the United States were recruited via Amazon’s Mechanical Turk (Buhrmeister et al., 2011) in exchange for monetary payment of $4.00. Participants’ mean age was 54.25 years (*SD* = 10.31). The racial composition of the sample was 85.8% White, 8.4% African American, 4.1% Asian American, and 1.8% other. Ethical approval was granted by the Partners Human Research Committee, and subjects provided written informed consent prior to participation.

### Stimulus Material

**FIGURE 1:**
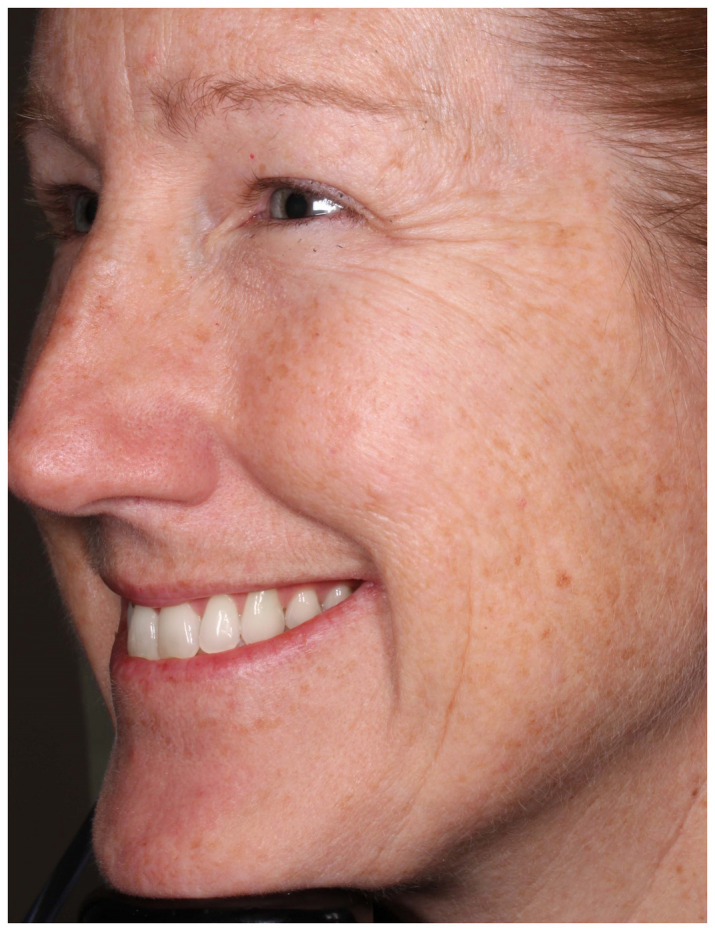
Participant before receiving botulinum toxin injection.

**FIGURE 2:**
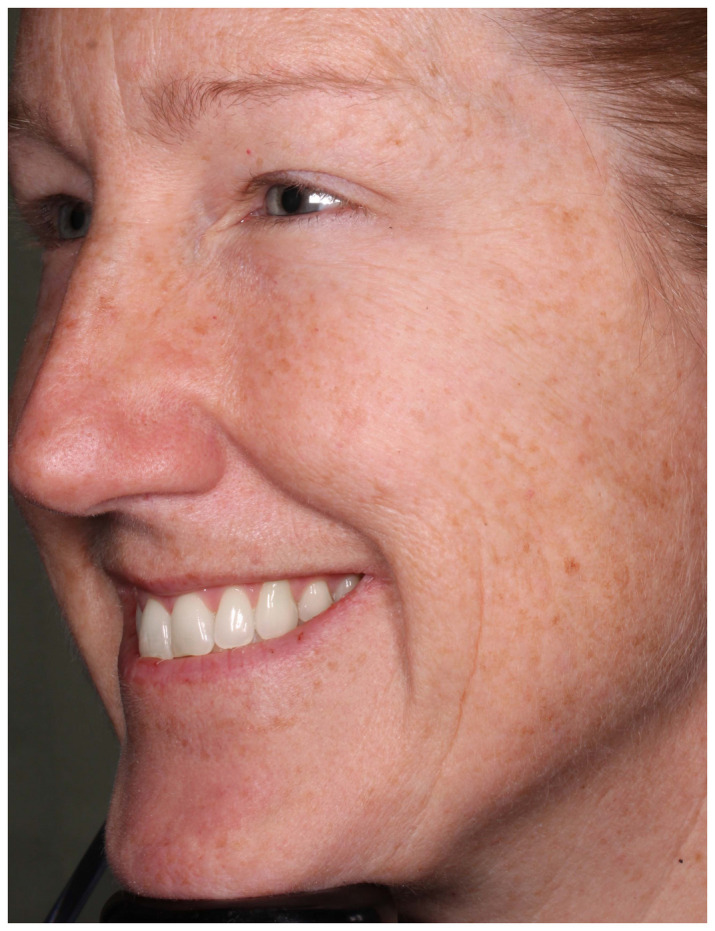
Participant after (approximately 1 month) receiving botulinum toxin injection.

The facial stimuli featured high resolution, full color images of five adult men and 27 women who had participated in a clinical trial examining the efficacy of botulinum toxin for the treatment of crow’s feet wrinkles (Carruthers et al., 2014). Each participant showed moderate or severe bilaterally symmetrical crow’s feet lines as assessed with the Facial Wrinkle Scale prior to treatment. Chemodenervation was applied by injecting botulinum toxin directly into the lateral orbicularis oculi muscle tissue to prevent muscular contraction in that focal area. All individuals (referred thereafter as patients) were asked to produce a “maximum” smile before and after (approximately 1 month) receiving the botulinum toxin injection. Specifically, they were told the following: “You should smile to show your biggest natural smile; you should not force the smile. You may smile with your lips parted or with your lips together, whichever feels more natural.” Each photograph was taken at an oblique (three-quarter viewing) angle using a standardized photographic apparatus with consistent lighting (see Figures 1, 2).

For all pre- and post-treatment images (64 images), a FACS certified coder who was blind to the treatment condition scored the presence of AU6 + 12 (the Duchenne marker) as well as AU12 intensity. Intercoder reliability was checked by a second FACS certified coder for 25% of the stimulus material (16 images). Mean agreement for the presence of AU 6 (Cohen’s Kappa (κ) = 0.875) and AU 12 (κ = 1.00) was high. All patients posing “maximum” smiles displayed AU12 activity before and after treatment. A Wilcoxon signed-rank test revealed no significant difference in AU12 intensity between pre-treatment (*M* = 3.19, *SD* = 0.69) and post-treatment images (*M* = 3.22, *SD* = 0.66), *Z* = 0.58, *p* = 0.564. Hence, images were well matched for smile intensity. When analyzing AU6 activity, the majority of participants (97%) were able to voluntarily contract the orbicularis oculi muscle prior to treatment. This fell to 19% after chemodenervation, which is a significant drop in the proportion from pre- to post-treatment (McNemar’s test, *p* < 0.001). As such, pre- and post-treatment photographs predominantly consisted of Duchenne (AU6 + 12) and non-Duchenne smiles (AU12), respectively.

### Procedure

Participants were tested individually using Qualtrics, a web-based software. After providing informed consent they were instructed that they would view a series of images of facial expressions. Their task was to rate each expression on several dimensions. Participants then viewed the 64 images (32 pre-treatment and 32 post-treatment) in a randomized order in three blocks. Each block showed the same photograph on screen for a duration of 10 s. The first block always started with the emotion rating scales. Here, participants responded to the query: “How much of the following emotions was the person feeling?” (happiness, sadness, anger, disgust, fear, surprise, and embarrassment). Ratings were made on 8-point Likert scales, with response options ranging from 0 (not at all/none) to 8 (extremely/a great deal).

In the second block, participants evaluated the smile quality of the expression by responding to the queries: “How felt was the expression of the person?” “How spontaneous was the expression of the person?” and “How intense was the expression of the person?” Ratings were made on 10-point Likert scales, with response options ranging from 0 (not at all/none) to 10 (extremely/a great deal). In the final block, participants rated the attractiveness of the person (0-not at all/none to 10-extremely/a great deal) and guessed the person’s age (from 19 and 99 years).

Participants completed the entire task in approximately 35 min. Although the experimental design allowed all 393 participants to rate each of the 32 pre-treatment and post-treatment image pairs, many stopped before viewing all images. On average, participants rated 7.86 pre-treatment and post-treatment photos, resulting in 3,090 observations at each time point.

## Results

To analyze the effect of treatment on each of these ratings, we used linear mixed-effects models with crossed random effects for patient and participant, and a fixed effect for treatment. Please see Baayen et al. (2008) for a detailed description of this modeling approach and Etcoff et al. (2011) for an example within the psychology literature on face perception.

The results of the linear mixed-effects models are summarized in Table 1. For emotion ratings, treatment was associated with a mean decrease of 0.11 in happiness ratings and a mean decrease of 0.07 in embarrassment ratings (*p* = 0.01 and *p* = 0.03 for treatment effects on happiness and embarrassment, respectively). There were no significant associations between treatment and any other measured emotions (sadness, anger, disgust, fear, or surprise).

**TABLE 1:** Mixed-effects models evaluating the effect of botulinum toxin treatment on ratings of émotions, smile, quality, attractiveness, and age.

Outcome	Covariate	Estimate	*SE*	*df*	*t*-stat	*p*
Happy	Intercept	4.441	0.14	33.6	31.51	< 0.0001
	Treatment (post vs. pre)	–0.111	0.05	5,757	–2.47	0.01
Sad	Intercept	0.482	0.04	42.3	12.64	< 0.0001
	Treatment (post vs. pre)	–0.035	0.03	5,764	–1.37	0.17
Anger	Intercept	0.307	0.03	43.6	10.10	< 0.0001
	Treatment (post vs. pre)	0.016	0.02	5,778	0.75	0.45
Disgust	Intercept	0.357	0.04	41	9.90	< 0.0001
	Treatment (post vs. pre)	0.026	0.02	5,782	1.14	0.25
Fear	Intercept	0.442	0.04	39.6	10.36	< 0.0001
	Treatment (post vs. pre)	0.001	0.03	5,766	0.03	0.98
Surprise	Intercept	1.087	0.07	40.7	16.48	< 0.0001
	Treatment (post vs. pre)	–0.004	0.04	5,775	–0.09	0.93
Embarrassment	Intercept	0.860	0.04	55.1	21.46	< 0.0001
	Treatment (post vs. pre)	–0.074	0.03	5,751	–2.20	0.03
Felt	Intercept	5.320	0.15	34.3	36.47	< 0.0001
	Treatment (post vs. pre)	–0.302	0.05	5,753	–5.79	< 0.0001
Spontaneity	Intercept	4.014	0.12	37.9	34.11	< 0.0001
	Treatment (post vs. pre)	–0.249	0.06	5,753	–4.06	< 0.0001
Intense	Intercept	4.374	0.18	33.7	24.61	< 0.0001
	Treatment (post vs. pre)	–0.239	0.06	5,757	–4.16	< 0.0001
Attract	Intercept	4.149	0.15	34.5	28.15	< 0.0001
	Treatment (post vs. pre)	–0.039	0.05	5,770	–0.74	0.46
Age	Intercept	42.836	0.91	32.1	46.84	< 0.0001
	Treatment (post vs. pre)	–0.937	0.19	5,758	–5.03	< 0.0001

Treatment was also associated with a statistically significant decrease in ratings of smile quality. Specifically, post-treatment photographs were associated with a mean decrease of 0.30 in felt ratings, 0.25 in spontaneity ratings, and 0.24 in intensity ratings (all treatment *p* < 0.001).

Treatment did not have a significant effect on ratings of attractiveness, although post-treatment photographs were associated with a mean age approximately 1 year younger than pre-treatment photographs (*p* < 0.001).

None of the statistically significant treatment effects were confounded by patient age, patient sex, participant age, or participant sex (see Supplementary Table 1). However, we did observe significant effects of patient sex on ratings of emotions and smile quality. Specifically, compared to males, photographs of female patients were associated with mean ratings 0.22 lower for sadness, 0.44 higher for surprise, 0.95 higher for felt, 0.73 higher for spontaneity, and 1.29 higher for intensity (all *p* ≤ 0.02). Ratings of female patients were also associated with a mean age approximately 3.7 years younger than males (*p* = 0.03). Among ratings of attractiveness, female participants rated images a mean of 0.23 higher than male participants (*p* < 0.001).

In a sensitivity analysis we reevaluated treatment effects after eliminating one patient without AU6 in their pre-treatment photograph and six patients with AU6 in their post-treatment photograph, which resulted in no substantive changes in our results (see Supplementary Table 2). Since our sample was imbalanced with respect to patient sex (27 females vs. 5 males), we also repeated our analyses among the subset of female patients. The only appreciable difference in these results was that treatment was no longer associated with a statistically significant effect on ratings of embarrassment (see Supplementary Table 3).

## Discussion

Virtually all pre-treatment photographs depicted patients displaying Duchenne smiles. These smiles were rated as being happier, more felt, more spontaneous, and more intense than those posed by the same patients under the same conditions and instructions in post-treatment photographs. The photographs were matched for smile intensity (activity of the zygomatic major muscle pulling the lip corner) suggesting that the differences were due to the inhibition of the orbicularis oculi and not by the activity of zygomatic major.

Patients were also rated as being significantly younger after treatment (by approximately 1 year) likely due to less visible crow’s feet lines. There was no effect of treatment on facial attractiveness ratings. Although some have speculated that a more spontaneous smile would make a face more attractive, past research (Mehu et al., 2007b) also found no difference in ratings of attractiveness for faces displaying Duchenne vs. non-Duchenne smiles. As such, attractiveness might be more dependent on the face structure and skin health than on dynamic features.

Interestingly, we found that ratings of smile quality were dependent on sex. The smiles of female patients were rated as more felt, surprised, spontaneous, and intense as well as less sad. This is consistent with data suggesting that women are more expressive for positive valanced facial actions (McDuff et al., 2017).

These data are consistent with results of previous studies demonstrating that Duchenne smiles are perceived differently than non-Duchenne smiles (Hess and Kleck, 1994; Del Giudice and Colle, 2007; Krumhuber and Manstead, 2009; Mehu et al., 2012; Gunnery et al., 2013). Patients in the pre-treatment photographs—consisting almost exclusively of Duchenne smiles—were perceived as feeling more genuine positive emotion in comparison to post-treatment photographs. These data are also consistent with studies reporting high frequencies of Duchenne smiles in deliberate facial action tasks (Kanade et al., 2000; Krumhuber et al., 2020). Together, these findings suggest that although the Duchenne marker can be posed in the absence of positive affect, it is still perceived by others to be indicative of genuine emotion. Future research may benefit from examining potential limitations in the production or inhibition of the Duchenne marker in facial action tasks. Such work could shed new light on how different elicitation conditions might drive the reliability of this signal (McCullough and Reed, 2016; see also Zloteanu et al., 2020 in the context of surprise expressions).

Our results have several potential implications and caveats. Our study did not support the strongest version of the Duchenne hypothesis—that inhibition of the orbicularis oculi would make the smile signal appear unfelt or weak. Non-Duchenne smiles were rated as less happy, genuine, felt, and spontaneous, though our small treatment effects suggest that the effect was subtle. However, our stimulus patients were instructed to pose “maximum smiles” (maximum zygomatic activation). It may be that more pronounced effects on smile authenticity occur with less intense smiles. In general, these small but statistically significant changes could have practical implications in natural contexts where smiles may be less intense and/or the Duchenne marker may be more conspicuous.

Previous research has shown that the Duchenne marker plays a role in communicating cooperative intent (Mehu et al., 2007a, b; Reed et al., 2012) as well as eliciting cooperation from others (Scharlemman et al., 2001; Brown and Moore, 2002). In light of the results of the current study, it is possible that when the Duchenne marker is absent (in this study through chemodenervation that inhibited the orbicularis oculi muscle and erased visible crow’s feet wrinkles) signals of cooperation may be lessened. If so, augmenting other signals of positive affect such as vocal affect or body language may counter the effects. Future studies can test this idea.

The images used in this study were derived from a clinical trial evaluating the efficacy of botulinum toxin on crow’s feet lines. In order to test our hypothesis, we selected a subset of patients who showed no evidence of crow’s feet lines using the Facial Wrinkle Scale post treatment. The majority of patients in the trial (66%), while having clinical improvement, did not have complete elimination of their dynamic lines. Interestingly, the patients in this trial where dynamic lines were eliminated reported feeling more satisfied with their appearance after treatment than those in whom some movement was preserved (unpublished data, Allergan). This suggests a potential disconnect between the positive perception of the aesthetic outcome on the part of the patient and the subtle negative impact on emotion communication as perceived by the observers. While not within the scope of this paper, this tension warrants further exploration. As reported, perceived smile authenticity did not impact attractiveness ratings, and did make patients appear approximately a year younger.

Three specific limitations must be taken into account when interpreting our findings. First, participants rated static images as opposed to video clips. Video clips have been shown to provide richer emotional content in comparison to static images (Ambadar et al., 2005; see Krumhuber et al., 2013, for a review) and would allow for the analysis of timing characteristics of facial expressions (Ambadar et al., 2009). Second, our sample did not include pre- and post-treatment photographs of spontaneously occurring smiles. That is, we were able to test our primary hypotheses using only deliberate facial action tasks and not when patients were experiencing genuine positive emotion (see Namba et al., 2020, for a similar approach). Future research addressing these limitations could complement the present findings and broaden our understanding of the perceptual and behavioral effects of the Duchenne smile. Third, our study was done solely with participants in the United States. It may be that participants in other cultures will be more impacted by the absence of the Duchenne marker. For example, Yuki et al. (2007) found cultural difference in the use of the eyes and mouth as cues to emotion in Japan and the U.S., with participants in Japan relying more heavily on eye expression for determination of emotion, including happiness, and participants in the U.S. on the mouth.

The Duchenne smile was first reported in 1862 by Duchenne de Boulogne in his “Mechanisme de la Physiognomie Humane.” Duchenne isolated facial muscle action using the novel method of electrical contraction of its muscles. These were the first physiological experiments illustrated by photography. Over 150 years later, we used a pharmacological technique to selectively chemodenervate, and therefore isolate specific facial muscles. In doing so, we shed further light on Duchenne’s pioneering ideas and address current controversies. We find evidence that Duchenne smiles communicate genuine and more intense happiness and that complete inhibition of orbicularis oculi leads to subtle yet statistically significant decreases in such communication.

## Data Availability Statement

The raw data supporting the conclusions of this article will be made available by the authors, without undue reservation, to any qualified researcher.

## Ethics Statement

The studies involving human participants were reviewed and approved by the Human Research Protection Program, the Institutional Review Board for all Mass General Brigham hospitals. The patients/participants provided their written informed consent to participate in this study. Written informed consent was obtained from the individual(s) for the publication of any potentially identifiable images or data included in this article.

## Author Contributions

NE developed the study concept and study design. LR, NE, and SS drafted the manuscript. EK provided critical revisions. SS did the data analysis. All authors did the data interpretation and approved the final version of the manuscript for submission.

## Conflict of Interest

This study was funded by Allergan, plc. The funders had no role in data collection, data analysis, or the decision to publish this manuscript. The authors declare that the research was conducted in the absence of any commercial or financial relationships that could be construed as a potential conflict of interest.
