# Supplementary material for: A Novel Test of the Duchenne Marker: Smiles After Botulinum Toxin Treatment for Crow’s Feet Wrinkles
Source: Front Psychol. 2021 Jan 12;11:612654. doi: 10.3389/fpsyg.2020.612654 (PMC7835207; doi:10.3389/fpsyg.2020.612654)
Supplement: Supplementary file 1 [file Table_1.DOCX]

**Supplementary Materials**

Table 1. Mixed-effects models evaluating the effect of botulinum toxin treatment on ratings of emotion, smile quality, attractiveness, and age after controlling for patient age, patient sex, rater age, and rater sex.

| Outcome | Covariate | Estimate | SE | df | t-stat | *p* |
| --- | --- | --- | --- | --- | --- | --- |
| Happy | Intercept | 4.48 | 0.89 | 30.8 | 5.01 | <.0001 |
|  | Treatment (post vs pre) | -0.11 | 0.05 | 5757 | -2.47 | 0.01 |
|  | Rater sex (female vs male) | 0.06 | 0.06 | 381 | 1.00 | 0.32 |
|  | Patient sex (female vs male) | 0.73 | 0.37 | 29 | 1.96 | 0.06 |
|  | Rater age | -0.001 | 0.0028 | 384 | -0.29 | 0.77 |
|  | Patient age | -0.01 | 0.0193 | 28.9 | -0.75 | 0.46 |
| Sad | Intercept | 0.75 | 0.23 | 37.2 | 3.21 | 0.003 |
|  | Treatment (post vs pre) | -0.03 | 0.03 | 5766 | -1.37 | 0.17 |
|  | Rater sex (female vs male) | -0.06 | 0.03 | 383 | -1.88 | 0.06 |
|  | Patient sex (female vs male) | -0.22 | 0.09 | 29.2 | -2.39 | 0.02 |
|  | Rater age | 0.0001 | 0.0015 | 388 | 0.07 | 0.94 |
|  | Patient age | -0.001 | 0.0048 | 28.8 | -0.28 | 0.78 |
| Anger | Intercept | 0.22 | 0.2 | 36.9 | 1.10 | 0.28 |
|  | Treatment (post vs pre) | 0.02 | 0.02 | 5779 | 0.75 | 0.45 |
|  | Rater sex (female vs male) | -0.03 | 0.02 | 398 | -1.23 | 0.22 |
|  | Patient sex (female vs male) | -0.03 | 0.08 | 29.3 | -0.41 | 0.69 |
|  | Rater age | -0.002 | 0.0012 | 403 | -1.57 | 0.12 |
|  | Patient age | 0.01 | 0.004 | 29 | 1.32 | 0.20 |
| Disgust | Intercept | 0.26 | 0.24 | 35.1 | 1.08 | 0.29 |
|  | Treatment (post vs pre) | 0.03 | 0.02 | 5782 | 1.14 | 0.25 |
|  | Rater sex (female vs male) | -0.03 | 0.03 | 402 | -1.07 | 0.28 |
|  | Patient sex (female vs male) | 0.04 | 0.09 | 29.1 | 0.45 | 0.65 |
|  | Rater age | -0.002 | 0.0013 | 407 | -1.56 | 0.12 |
|  | Patient age | 0.004 | 0.0049 | 28.9 | 0.87 | 0.39 |
| Fear | Intercept | 0.35 | 0.28 | 34.2 | 1.25 | 0.22 |
|  | Treatment (post vs pre) | 0 | 0.03 | 5765 | 0.03 | 0.98 |
|  | Rater sex (female vs male) | 0 | 0.03 | 383 | 0.01 | 1 |
|  | Patient sex (female vs male) | 0.1 | 0.11 | 29 | 0.92 | 0.37 |
|  | Rater age | -0.002 | 0.0014 | 388 | -1.50 | 0.14 |
|  | Patient age | 0.003 | 0.0059 | 28.8 | 0.48 | 0.64 |
| Surprise | Intercept | 0.11 | 0.38 | 37.3 | 0.29 | 0.78 |
|  | Treatment (post vs pre) | -0.0036 | 0.04 | 5776 | -0.09 | 0.93 |
|  | Rater sex (female vs male) | -0.02 | 0.05 | 398 | -0.4 | 0.69 |
|  | Patient sex (female vs male) | 0.44 | 0.15 | 29.1 | 2.95 | 0.01 |
|  | Rater age | 0.002 | 0.0024 | 402 | 0.78 | 0.44 |
|  | Patient age | 0.01 | 0.0077 | 28.7 | 1.54 | 0.14 |
| Embarrassment | Intercept | 1.03 | 0.26 | 42.9 | 3.97 | <.0001 |
|  | Treatment (post vs pre) | -0.07 | 0.03 | 5752 | -2.20 | 0.03 |
|  | Rater sex (female vs male) | -0.04 | 0.04 | 372 | -1.04 | 0.30 |
|  | Patient sex (female vs male) | -0.04 | 0.1 | 29.2 | -0.43 | 0.67 |
|  | Rater age | -0.003 | 0.002 | 376 | -1.44 | 0.15 |
|  | Patient age | 0.001 | 0.0051 | 28.6 | 0.18 | 0.86 |
| Felt | Intercept | 4.46 | 0.89 | 31.4 | 5.01 | <.0001 |
|  | Treatment (post vs pre) | -0.30 | 0.05 | 5754 | -5.79 | <.0001 |
|  | Rater sex (female vs male) | 0.08 | 0.07 | 379 | 1.16 | 0.25 |
|  | Model sex (female vs male) | 0.95 | 0.37 | 29 | 2.59 | 0.02 |
|  | Rater age | 0.004 | 0.0033 | 383 | 1.25 | 0.21 |
|  | Patient age | -0.005 | 0.0191 | 28.9 | -0.24 | 0.81 |
| Spontaneity | Intercept | 3.06 | 0.72 | 33.9 | 4.27 | 0 |
|  | Treatment (post vs pre) | -0.25 | 0.06 | 5755 | -4.06 | <.0001 |
|  | Rater sex (female vs male) | 0.01 | 0.08 | 378 | 0.09 | 0.93 |
|  | Patient sex (female vs male) | 0.73 | 0.29 | 28.9 | 2.52 | 0.02 |
|  | Rater age | 0.01 | 0.0037 | 382 | 2.04 | 0.04 |
|  | Patient age | -0.002 | 0.0151 | 28.7 | -0.11 | 0.92 |
| Intense | Intercept | 2.77 | 1.05 | 31.2 | 2.63 | 0.01 |
|  | Treatment (post vs pre) | -0.24 | 0.06 | 5759 | -4.16 | <.0001 |
|  | Rater sex (female vs male) | -0.1 | 0.07 | 384 | -1.37 | 0.17 |
|  | Patient sex (female vs male) | 1.29 | 0.43 | 29.1 | 2.97 | 0.01 |
|  | Rater age | 0.01 | 0.0036 | 387 | 1.90 | 0.06 |
|  | Patient age | 0.004 | 0.0226 | 29 | 0.20 | 0.85 |
| Attract | Intercept | 5.3 | 0.93 | 31.3 | 5.70 | <.0001 |
|  | Treatment (post vs pre) | -0.04 | 0.05 | 5770 | -0.74 | 0.46 |
|  | Rater sex (female vs male) | 0.23 | 0.07 | 393 | 3.40 | <.001 |
|  | Patient sex (female vs male) | 0.3 | 0.38 | 29 | 0.77 | 0.45 |
|  | Rater age | 0.005 | 0.0033 | 396 | 1.54 | 0.12 |
|  | Patient age | -0.04 | 0.02 | 28.9 | -2.04 | 0.05 |
| Age | Intercept | 20.46 | 3.99 | 30.6 | 5.13 | <.0001 |
|  | Treatment (post vs pre) | -0.94 | 0.19 | 5759 | -5.03 | <.0001 |
|  | Rater sex (female vs male) | -0.09 | 0.24 | 383 | -0.39 | 0.7 |
|  | Patient sex (female vs male) | -3.67 | 1.66 | 29.1 | -2.22 | 0.03 |
|  | Rater age | 0.03 | 0.0116 | 386 | 2.31 | 0.02 |
|  | Patient age | 0.54 | 0.0861 | 29 | 6.35 | <.0001 |

Table 2. Mixed-effects models evaluating the effect of botulinum toxin treatment on ratings of emotion, smile quality, attractiveness, and age. Analyses were based on the subset of models with AU6 pre-treatment and without AU6 post-treatment.

| Outcome | Covariate | Estimate | SE | df | t-stat | *p* |
| --- | --- | --- | --- | --- | --- | --- |
| Happy | Intercept | 4.493 | 0.15 | 26.3 | 29.43 | <.0001 |
|  | Treatment (post vs pre) | -0.136 | 0.05 | 4393.0 | -2.71 | 0.01 |
| Sad | Intercept | 0.454 | 0.04 | 32.9 | 11.25 | <.0001 |
|  | Treatment (post vs pre) | -0.020 | 0.03 | 4409.0 | -0.71 | 0.48 |
| Anger | Intercept | 0.289 | 0.03 | 43.4 | 10.48 | <.0001 |
|  | Treatment (post vs pre) | 0.029 | 0.02 | 4431.0 | 1.26 | 0.21 |
| Disgust | Intercept | 0.327 | 0.04 | 34.8 | 9.26 | <.0001 |
|  | Treatment (post vs pre) | 0.034 | 0.02 | 4425.0 | 1.38 | 0.17 |
| Fear | Intercept | 0.436 | 0.04 | 33.5 | 10.55 | <.0001 |
|  | Treatment (post vs pre) | 0.008 | 0.03 | 4408.0 | 0.29 | 0.77 |
| Surprise | Intercept | 1.084 | 0.08 | 30.5 | 14.22 | <.0001 |
|  | Treatment (post vs pre) | 0.010 | 0.04 | 4417.0 | 0.23 | 0.82 |
| Embarrassment | Intercept | 0.870 | 0.05 | 40.7 | 18.62 | <.0001 |
|  | Treatment (post vs pre) | -0.090 | 0.04 | 4403.0 | -2.35 | 0.02 |
| Felt | Intercept | 5.321 | 0.16 | 26.6 | 32.47 | <.0001 |
|  | Treatment (post vs pre) | -0.321 | 0.06 | 4391.0 | -5.47 | <.0001 |
| Spontaneity | Intercept | 4.029 | 0.13 | 29.2 | 30.31 | <.0001 |
|  | Treatment (post vs pre) | -0.275 | 0.07 | 4398.0 | -3.99 | <.0001 |
| Intense | Intercept | 4.366 | 0.20 | 26.1 | 21.64 | <.0001 |
|  | Treatment (post vs pre) | -0.244 | 0.06 | 4399.0 | -3.76 | 0.0002 |
| Attract | Intercept | 4.200 | 0.18 | 26.6 | 23.84 | <.0001 |
|  | Treatment (post vs pre) | -0.030 | 0.06 | 4403.0 | -0.49 | 0.62 |
| Age | Intercept | 42.536 | 1.06 | 24.7 | 40.19 | <.0001 |
|  | Treatment (post vs pre) | -1.105 | 0.21 | 4410.0 | -5.30 | <.0001 |

Table 3. Mixed-effects models evaluating the effect of botulinum toxin treatment on ratings of emotion, smile quality, attractiveness, and age among female patients.

| Outcome | Covariate | Estimate | *SE* | df | t-stat | *p* |
| --- | --- | --- | --- | --- | --- | --- |
| Happy | Intercept | 4.545 | 0.16 | 28 | 29.06 | <.0001 |
|  | Treatment (post vs pre) | -0.100 | 0.05 | 4828 | -2.04 | 0.0418 |
| Sad | Intercept | 0.436 | 0.04 | 35.4 | 11.48 | <.0001 |
|  | Treatment (post vs pre) | -0.014 | 0.03 | 4863 | -0.52 | 0.6011 |
| Anger | Intercept | 0.308 | 0.04 | 34.5 | 8.71 | <.0001 |
|  | Treatment (post vs pre) | 0.007 | 0.02 | 4849 | 0.32 | 0.7481 |
| Disgust | Intercept | 0.360 | 0.04 | 32.8 | 8.66 | <.0001 |
|  | Treatment (post vs pre) | 0.036 | 0.03 | 4852 | 1.43 | 0.1538 |
| Fear | Intercept | 0.453 | 0.05 | 32.2 | 9.18 | <.0001 |
|  | Treatment (post vs pre) | 0.013 | 0.03 | 4841 | 0.45 | 0.6539 |
| Surprise | Intercept | 1.160 | 0.07 | 36.2 | 17.24 | <.0001 |
|  | Treatment (post vs pre) | -0.004 | 0.04 | 4837 | -0.09 | 0.9305 |
| Embarrassment | Intercept | 0.841 | 0.04 | 45.3 | 18.99 | <.0001 |
|  | Treatment (post vs pre) | -0.049 | 0.04 | 4824 | -1.35 | 0.1771 |
| Felt | Intercept | 5.457 | 0.16 | 28.8 | 35.19 | <.0001 |
|  | Treatment (post vs pre) | -0.281 | 0.06 | 4832 | -4.92 | <.0001 |
| Spontaneity | Intercept | 4.109 | 0.12 | 32.7 | 33.62 | <.0001 |
|  | Treatment (post vs pre) | -0.209 | 0.07 | 4825 | -3.14 | 0.0017 |
| Intense | Intercept | 4.569 | 0.18 | 28.6 | 24.95 | <.0001 |
|  | Treatment (post vs pre) | -0.219 | 0.06 | 4828 | -3.47 | 0.0005 |
| Attract | Intercept | 4.195 | 0.16 | 28.7 | 25.93 | <.0001 |
|  | Treatment (post vs pre) | -0.063 | 0.06 | 4842 | -1.10 | 0.2711 |
| Age | Intercept | 42.408 | 0.99 | 26.9 | 42.69 | <.0001 |
|  | Treatment (post vs pre) | -0.868 | 0.20 | 4828 | -4.25 | <.0001 |
